# Associations of Serum Retinol, α-Tocopherol, and γ-Tocopherol with Biomarkers among Healthy Japanese Men 

**DOI:** 10.3390/ijerph110201647

**Published:** 2014-01-30

**Authors:** Yu Zou, Da-Hong Wang, Noriko Sakano, Yoshie Sato, Suketaka Iwanaga, Kazuhisa Taketa, Masayuki Kubo, Kei Takemoto, Chie Masatomi, Kiyomi Inoue, Keiki Ogino

**Affiliations:** 1 Department of Public Health, Graduate School of Medicine, Dentistry and Pharmaceutical Sciences, Okayama University, 2-5-1 Shikata-cho, Okayama 700-8558, Japan; E-Mails: rainyjo333@gmail.com (Y.Z.); masakubo@cc.okayama-u.ac.jp (M.K.); kei_takemoto@okayama-u.ac.jp (K.T.); ma.sa.chi.n.0409@gmail.com (C.M.); kogino@md.okayama-u.ac.jp (K.O.); 2 Department of Gerontology Research, Graduate School of Medicine, Dentistry and Pharmaceutical Sciences, Okayama University, 2-5-1 Shikata-cho, Okayama 700-8558, Japan; E-Mail: snoriko@cc.okayama-u.ac.jp; 3 Graduate School of Health Sciences, Okayama University, 2-5-1 Shikata-cho, Okayama 700-8558, Japan; E-Mail: yoshie@md.okayama-u.ac.jp; 4 Department of Public Health, Faculty of Medicine, Kyoto University, Yoshida-Konoye-cho Sakyo-ku, Kyoto 606-8501, Japan; E-Mail: iwanaga.at.work@gmail.com; 5 Geriatric Health Service Facility, Niwanosato Home, Mihara, Hiroshima 729-1321, Japan; E-Mail: taketa@rijinkai.or.jp; 6 Public Health Care Nursing, Department of Nursing, Faculty of Health Sciences, Kobe Tokiwa University, 2-6-2 Otani-cho, Kobe 653-0838, Japan; E-Mail: k-inoue@kobe-tokiwa.ac.jp

**Keywords:** serum retinol, serum α-tocopherol, serum γ-tocopherol, HOMA-R, serum ferritin, smoking, alcohol consumption

## Abstract

Retinol, α-tocopherol, and γ-tocopherol are fat-soluble vitamins acting as antioxidants via the prevention of lipid oxidation. Little is known about circulatory levels in healthy individuals. The present cross-sectional study aimed at elucidating the relationship between these antioxidants and clinical biomarkers in 206 male (median age 41 years, range 23–67) employees from companies located in the Okayama Prefecture, Japan. Subjects younger than 40 years (n = 94) showed a positive association of the frequency of alcohol consumption with the circulating retinol (β = 0.344, *p* = 0.001) and γ-tocopherol levels (β = 0.219, *p* = 0.041), and an inverse association of fast insulin with serum retinol (β = −0.301, *p* = 0.009). In participants older than 40 years (n = 112) we found that an inverse association of HOMA-R with serum retinol (β = −0.262, *p* = 0.021), α-tocopherol (β = −0.236, *p* = 0.035), and γ-tocopherol levels (β = −0.224, *p* = 0.052); and cigarette smoking was inversely associated with the levels of serum α-tocopherol (β = −0.286, *p* = 0.008) and γ-tocopherol (β = −0.229, *p* = 0.040). We further found negative relationships between serum ferritin and the retinol (β = −0.211, *p* = 0.032) and α-tocopherol levels (β = −0.223, *p* = 0.022) in men over 40 years of age. The present study suggests that the circulatory levels of antioxidant vitamins may modulate the action of insulin and that higher levels of iron might decrease the levels of antioxidant vitamins in the blood.

## 1. Introduction

Many studies have demonstrated that oxidative stress plays an important role in the pathogenesis of various chronic diseases, such as diabetes, cancer, and cardiovascular diseases [1], and it has been suggested that antioxidants play a protective role against various diseases by defending against oxidative damage [2,3,4]. Measurements of circulating vitamin levels have been used to assess the antioxidant status in humans [5], although many researchers have focused on examining the circulating vitamin levels in diseased persons [6,7,8]. How the circulatory levels of antioxidant vitamins within a healthy population associate with clinical biomarkers has not been fully elucidated.

Retinol, α-tocopherol, and γ-tocopherol are fat-soluble vitamins that require protein carriers for transportation in the blood. Retinol is one of the active forms of vitamin A; it can promote vision, participate in protein synthesis and cell differentiation, support reproduction and growth [9], and inhibit lipid peroxidation [10]. The two main forms of vitamin E are α-tocopherol and γ-tocopherol; the concentrations of α-tocopherol are reported to be 4–10 times higher than those of γ-tocopherol in the plasma among humans [11]. They both exert an antioxidant action via the prevention of lipid oxidation, but α-tocopherol is considered to be more powerful relative to γ-tocopherol for inhibiting lipid peroxidation [12], and it has been suggested that γ-tocopherol is superior in detoxifying reactive nitrogen oxide species [13]. The present study aimed to examine the relationships of serum retinol, α-tocopherol, γ-tocopherol with biomarkers among healthy men in Japan.

## 2. Subjects and Methods

### 2.1. Participants

The data are based on a worksite lifestyle intervention study in a prefecture of Japan that included 847 individuals (360 men, 487 women) from six companies who participated from September to December of 2007. The current study was restricted to male participants. After excluding the subjects who reported any supplement or medicine use or any history of diabetes, hyperlipaemia, cancer, stroke, or ischemic heart disease, the final data analysis was carried out for 206 men. The study was approved by the Ethics Committee of Okayama University Graduate School of Medicine, Dentistry, and Pharmaceutical Sciences. Written informed consent was obtained from all participants.

### 2.2. Measurement of Health Parameters and Oxidative Biomarkers

All anthropometric parameters were measured by the staffs of a local health management center. Body composition was evaluated using the following parameters: body weight; waist circumference; and body mass index (BMI), which was calculated by the body weight (kg)/height (m^2^). Each subject had his blood pressure measured by a physician with the subject in a sitting position after resting for at least a few minutes.

Venous blood and urine samples were collected after an overnight fast of at least 10 h. Serum and plasma were used to measure the total cholesterol (TC), triglycerides (TG), high density lipoprotein-cholesterol (HDL-c), low density lipoprotein-cholesterol (LDL-c), high-sensitivity C-reactive protein (hs-CRP), fasting glucose, insulin, haemoglobin A1c (HbA1c), serum Fe, and ferritin. The homeostasis model assessment (HOMA-R) levels were calculated as the fasting insulin (μU/mL) × fasting glucose (mg/dL)/405. The NOx levels (NO_2_^−^ + NO_3_^−^) in the serum were determined using a NO analyser (model-280i NOA with the Purge Vessel; Sievers, Boulder, CO, USA). The levels of urinary hydrogen peroxide (H_2_O_2_), 8-hydroxy-2′-deoxyguanosine (8-OHdG), and 8-isoprostane were determined from spot urine samples stored at −80 °C until analysis. The urinary H_2_O_2_ was analysed using the ferrous ion oxidation xylenol orange version-1 (FOX-1) assay. The measurement of 8-OHdG was carried out using an enzyme-linked immunosorbent assay (ELISA) kit (Japan Institute for the Control of Aging, Fukuroi, Shizuoka, Japan). The urinary 8-isoprostane levels were determined using a commercially available competitive enzyme immunoassay (EIA) kit (Cayman Chemical Company, Ann Arbor, MI, USA) Detailed descriptions of the measurements of these oxidative stress biomarkers have been included in previous reports [14,15].

### 2.3. Measurement of Serum Antioxidant Vitamins

A previous study has indicated that vitamins A and E in serum stored at −70 °C remain stable for a long period of time [16,17]. In the present study, fasting serum concentrations of retinol and α- and γ-tocopherol were measured in stored samples (frozen at −80 °C) using high-performance liquid chromatography paired with a diode array detector (Hitachi L-2455; Hitachi Ltd., Tokyo, Japan) (HPLC-DAD) [6]. The separation was carried out on an Inertsil ODS-3 column (5.0 µm, 25 cm × 4.6 mm I.D.) by elution with methanol: water (97:3, vol/vol) at a flow rate of 1.0 mL/min. The effluents were monitored simultaneously at 325 nm for retinol, 292 nm for α-tocopherol, and 297 nm for γ-tocopherol. Standard retinol, α-tocopherol and γ-tocopherol were obtained from Sigma-Aldrich, Inc., (St. Louis, MO, USA). The inter-assay coefficients of variation were 9.5%, 10.5%, and 11.7% for serum retinol, α-tocopherol, and γ-tocopherol, respectively.

### 2.4. Information on Lifestyles

Lifestyle data, including diet, cigarette smoking, alcohol consumption, and exercise, were obtained using self-reported questionnaires. Smoking status was classified into two groups: nonsmokers and current smokers. Alcohol consumption was converted into number of units per week. One unit was considered to be equivalent to 9–12 g for ethanol. Frequency of alcohol consumption was classified into three groups: non-drinking, less than 4 times per week and 4 times or above per week. The habit of exercise was defined as no exercise, 2 times per week or below, 3–5 times per week, and more than 5 times per week. 

Dietary information was assessed using a validated semi-quantitative food frequency questionnaire (FFQ) according to food groups. Participants were asked to specify, for the previous 1–2 months, how often, on average, they consumed the foods in each food group in a week, either as indicated by the unit and portion size or by scoring the consumption status on a 4-point Likert scale consisting of the responses “not at all”, “somewhat” (half of the general amount), “a general amount” (the amount consumed by people of the same sex and age), and “very much” (1.5 times the general amount). The questionnaire was validated by Takahashi *et al.* [18]. We used the residual method to adjust the nutrient intake information according to the total energy intake by performing a regression analysis [19].

### 2.5. Statistical Analysis

Group comparisons were carried out using the Mann-Whitney *U* test for the continuous variables and the χ² test for the categorical variables. The correlations of serum retinol, α-tocopherol, and γ-tocopherol with clinical biomarkers and estimated dietary nutrient intakes were examined using Spearman’s correlation coefficients. We also employed multiple regression analysis to explore how the serum antioxidative vitamin status (adjusted for the triglyceride level) was associated with the clinical biomarkers and dietary antioxidants, and the Durbin-Watson statistic was calculated to examine whether a residual was distributed normally. The relationships between serum retinol, α-tocopherol, and γ-tocopherol and clinical biomarkers were statistically analysed after making adjustments for the serum TG, as these fat-soluble vitamins are transported by the chylomicrons (which mainly contain TG) in the blood [20]. The variables with skewed distributions were log-transformed. All statistical analyses were carried out using SPSS (Statistical Package for the Social Sciences) 15.0 for Windows (SPSS Inc., Chicago, IL, USA).

## 3. Results and Discussion

Table 1 summarises the characteristics of the participants. The median age of the participants was 41 years, with a range from 23 to 67 years. Of the participants, 38% were current smokers, and 83% reported alcohol consumption. The participants over 40 years of age presented higher values of BMI, waist circumference, blood pressure, serum retinal, α-tocopherol, γ-tocopherol, total cholesterol, triglyceride, glucose, HbA1c and urinary 8-OHdG relative to those under 40. There was no difference in the daily nutrient intake between those under 40 and those over 40 years of age (Table 2). We did not find any significant correlation of serum retinol, α-tocopherol, and γ-tocopherol with the nutrient intake (Data not shown).

**Table 1:** Characteristics and clinical profile of subjects.

Variable	Median (min, max)			*p* ^b^
	All Subjects (n = 206)	Age < 40 (n = 94)	Age ≥ 40 (n = 112)	
Age (year)	41 (23, 67)			
BMI(kg/m^2^)	23.6 (16.1, 37.2)	23.5 (16.1, 35.8)	23.6 (16.5, 37.2)	0.576
Waist circumference (cm)	83.5 (61.5, 118.2)	81.3 (61.5, 118.2)	84 (67.1, 113)	0.010
Systolic blood pressure (mmHg)	131 (99, 193)	128 (101, 184)	135.5 (99, 193)	0.004
Diastolic blood pressure (mmHg)	81 (52, 124)	77 (53, 124)	82 (52, 121)	0.016
Smoker **^a^**	78 (37.9)	37 (39.4)	41 (36.6)	0.004
Alcohol drinking **^a^**				<0.001
No	36 (17.5)	18 (19.1)	18 (16.1)	
<4 times/week	84 (40.8)	52 (55.3)	32 (28.6)	
≥4 times/week	86 (41.7)	24 (25.5)	62 (55.4)	
Exercise **^a^**				0.958
No	91 (44.2)	42 (44.7)	49 (43.8)	
≤2 times/week	71 (34.4)	31 (32.9)	40 (35.7)	
3–5 times/week	29 (14.1)	15 (16.0)	14 (12.5)	
>5 times/week	25 (7.3)	6 (6.4)	9 (8.0)	
Serum retinol (mg/L)	1.3 (0.0, 2.8)	1.2 (0.5, 2.6)	1.4 (0.0, 2.8)	0.001
Serum α-tocopherol (mg/L)	6.5 (1.7, 15.4)	6.5 (1.7, 13.7)	6.9 (1.7, 15.4)	0.005
Serum γ-tocopherol (mg/L)	0.5 (0.1, 4.2)	0.5 (0.1, 1.7)	0.6 (0.2, 4.2)	0.003
TC (mg/dL)	204 (128, 284)	193 (128, 284)	209.5 (137, 277)	0.010
HDL-c (mg/dL)	57 (35, 133)	56 (35, 119)	59 (35, 133)	0.259
LDL-c (mg/dL)	128.5 (53, 209)	123.5 (53, 209)	130 (64, 195)	0.299
TG (mg/dl)	99.5 (30, 600)	85 (30, 349)	110.5 (33, 600)	0.025
Uric acid (mg/dL)	6.0 (2.8, 9.2)	6.1 (2.8, 9.0)	6.0 (3.4, 9.2)	0.998
Insulin (μU/mL)	4.4 (0.5, 30.4)	4.4 (0.5, 15.5)	4.4 (1.1, 30.4)	0.727
Fasting glucose (mg/dL)	92 (68, 169)	89.5 (68, 140)	94 (79, 169)	<0.001
HbA1c (%)	4.9 (3.7, 7.4)	4.9 (3.7, 7.1)	4.9 (4.2, 7.4)	0.012
HOMA-R	1.0 (0.1, 8.7)	1.0 (0.1, 3.5)	1.0 (0.3, 8.7)	0.735
Hs-CRP (mg/dL)	0.3 (0.0, 46.5)	0.3 (0.0, 46.5)	0.4 (0.1, 7.4)	0.767
Serum Fe (μg/dL)	112 (10, 273)	118 (31, 273)	111 (10, 214)	0.413
Ferritin (ng/ml)	165.2 (2.8, 785.1)	149.8 (9.3, 712.8)	171.1 (2.8, 785.1)	0.076
NO_x_ (μmol/L)	25.8 (8.6, 112.4)	25.1 (8.6, 102.6)	26.4 (8.9, 112.4)	0.464
H_2_O_2_ (μmol/g creatinine)	3.0 (0.0, 51.4)	2.9 (0.0, 41.3)	3.2 (0.0, 51.4)	0.304
8-OHdG (ng/mg creatinine)	8.5 (2.1, 21.7)	7.8 (2.7, 21.7)	9.1 (2.1, 19.6)	0.028
8-Isoprostane (pg/mg creatinine)	654.0 (6.6, 4,372.9)	658.4 (7.5, 3,408.3)	640.4 (6.6, 4,372.9)	0.945

Notes: BMI, body mass index; TC, total cholesterol; LDL-c, low-density lipoprotein cholesterol; HDL-c, high-density lipoprotein cholesterol; TG, triglycerides; HbA1c, haemoglobin A1c; HOMA-R, homeostasis model assessment ratio; Hs-CRP, high-sensitivity C-reactive; protein; NO_x_, nitrogen oxide; H_2_O_2_, hydrogen peroxide; 8-OHdG, 8-hydroxy-2’-deoxyguanosine; **^a^** n (%); **^b^** Mann-Whitney *U* test or χ^2^ test.

**Table 2:** Daily nutrient intakes of the subjects.

Variable	Median (min, max)			*p* ^a^
	All Subjects	Age < 40	Age ≥ 40	
Energy (Kcal)	1,796 (587, 6,999)	1,818 (746, 6,999)	1,774 (587, 3,192)	0.626
Protein (g)	59 (15, 186)	58 (22, 186)	60 (15, 114)	0.775
Fat (g)	57 (15, 344)	59.8 (26, 344)	56.1 (15, 109)	0.135
Carbohydrate (g)	233 (57, 771)	235 (71, 771)	232 (57, 447)	0.610
Sodium (mg)	3,361.5 (1,134, 9,707)	3,194 (1,134, 9,707)	3,559 (1,481, 7,722)	0.032
Potassium (mg)	1,800.5 (556, 5,120)	1,817 (668, 4,518)	1,795 (556, 5,120)	0.578
Calcium (mg)	419 (105, 5,173)	444 (167, 5,173)	405 (105, 1,156)	0.463
Magnesium (mg)	198 (54, 529)	199.5 (66, 529)	194.5 (54, 492)	0.932
Phosphorus (mg)	853.5 (244, 2,080)	821.5 (277, 2,080)	856.5 (244, 1,824)	0.805
Iron (mg)	6.1 (2.1, 100.4)	6.2 (2.2, 100.4)	6.0 (2.1, 13.8)	0.506
Zinc (mg)	7.0 (1.6, 16.1)	6.9 (2.2, 16.1)	7.1 (1.6, 14.7)	0.913
Copper (mg)	0.9 (0.3, 2.7)	0.9 (0.3, 2.7)	0.9 (0.3, 2.3)	0.981
Manganese (mg)	2.1 (0.5, 5.6)	2.1 (0.5, 4.7)	2.2 (0.5, 5.6)	0.569
Retinol (μg)	181 (25, 469)	180.5 (69, 438)	181 (25, 469)	0.359
α-Carotene (μg)	346.5 (1, 1,999)	315 (4, 1,999)	346.5 (1, 1,032)	0.711
β-Carotene (μg)	2,096.5 (20, 11,649)	1,997.5 (64, 11,649)	2,221 (20, 6,101)	0.646
β-Cryptoxanthin (μg)	214.5 (6, 2,815)	200 (17, 2,558)	220 (6, 2,815)	0.464
Vitamin D (μg)	5.6 (0.7, 19.9)	5.2 (0.8, 19.9)	6.0 (0.7, 15.8)	0.306
α-Tocopherol (mg)	5.5 (2.0, 15.2)	5.7 (2.0, 15.2)	5.4 (2.1, 11.5)	0.392
β-Tocopherol (mg)	0.3 (0.1, 0.7)	0.3 (0.1, 0.7)	0.3 (0.1, 0.7)	0.384
γ-Tocopherol (mg)	9.8 (2.1, 32.8)	9.9 (3.0, 32.8)	9.7 (2.1, 24.8)	0.681
δ-Tocopherol (mg)	2.7 (0.7, 6.6)	2.6 (1.0, 6.6)	2.7 (0.8, 6.0)	0.837
Vitamin K (μg)	142 (18, 499)	136 (18, 499)	145.5 (24, 384)	0.630
Vitamin B-1 (mg)	0.8 (0.2, 11.8)	0.8 (0.3, 11.8)	0.7 (0.2, 1.8)	0.241
Vitamin B-2 (mg)	0.9 (0.3, 7.7)	0.9 (0.4, 7.7)	0.9 (0.3, 2.2)	0.356
Niacin (mg)	14.1 (1.8, 35.3)	14.1 (4.2, 32.1)	14 (1.8, 35.3)	0.914
Vitamin B-6 (mg)	0.9 (0.1, 2.1)	0.9 (0.3, 2.1)	1.0 (0.1, 2.1)	0.316
Vitamin B-12 (μg)	5.6 (0.4, 17.0)	5.4 (1.1, 17.0)	5.9 (0.4, 15.4)	0.204
Folate (μg)	186 (46, 576)	178 (70, 576)	192 (46, 534)	0.562
Pantothenic acid (mg)	4.5 (1.5, 11.1)	4.5 (1.6, 11.1)	4.6 (1.5, 10.4)	0.493
Vitamin C (mg)	52.5 (4, 491)	52 (13, 491)	54 (4, 250)	0.878

Note: ^**a**^ Mann-Whitney *U* test.

### 3.1. Antioxidant Vitamins in Circulation May Modulate the Action of Insulin

Both the Spearman’s correlation coefficients and the multivariate-adjusted regression models revealed an inverse association of HOMA-R with the serum retinol, α-tocopherol, and γ-tocopherol levels in men, particularly in those over 40 (Table 3, Table 4, Table 5 and Table 6), and HbA1c was also inversely related to the level of serum γ-tocopherol (Table 3, Table 6). An early cohort study in Finland found that men with low plasma vitamin E were at an increased risk of non-insulin-dependent diabetes [21]. In alloxan-induced diabetic mice, Kamimura *et al.* [22] observed that the incidence of hyperglycaemia was higher in the mice maintained on the vitamin E-deprived diet than in those maintained on the vitamin E-supplemented diet. Because pancreatic islet cells express the antioxidant enzyme gene at a lower level, they are more sensitive to reactive oxygen species (ROS). It is known that ROS are involved in insulin resistance [23]. Higher circulating antioxidant vitamins could protect pancreatic islet cells against ROS, most likely contributing to a lowering of the insulin resistance.

We further found an inverse association between fast insulin and serum retinol (Table 3 and Table 4). The finding is inconsistent with that of Blondin *et al.* [24], although the latter research was targeted at young women. It is known that retinol in the blood is transported by a carrier called retinol-binding protein (RBP) [25]. Some researchers consider RBP to be an indicator of the vitamin A status [26]. Quadro *et al.* [27] found impaired retinol availability in the blood of mice lacking RBP. It has also been reported that a RBP receptor known as STRA6 either acts as a major physiological mediator of cellular retinol uptake or is related to the insulin response [28]. Accordingly, it appears that the direction of association between fast insulin and serum retinol is likely influenced by the actions of retinol-binding protein and its receptors.

**Table 3:** Spearman’s correlation of serum vitamins with each parameter.

Variable	Serum Retinol ^3^		Serum α-Tocopherol ^3^		Serum γ-Tocopherol ^3^	
	r	*p*	r	*p*	r	*p*
Age (years)	−0.059	0.401	−0.081	0.250	−0.046	0.513
BMI	**−0.236**	0.001	−**0.213**	0.002	−0.189	0.006
Cigarette **^1^**	−0.027	0.784	−0.087	0.376	−0.103	0.294
Alcohol consumption (unit/times) **^2^**	0.039	0.578	−0.065	0.352	0.034	0.626
Waist circumference	**−0.228**	0.001	**−0.212**	0.002	−0.148	0.034
Systolic blood pressure	−0.097	0.165	−0.096	0.173	−0.072	0.304
Diastolic blood pressure	−0.157	0.025	−0.124	0.078	−0.077	0.270
TC	**−0.298**	<0.001	−0.146	0.037	−0.165	0.017
HDL-c	**0.446**	<0.001	**0.444**	<0.001	**0.394**	<0.001
LDL-c	**−0.267**	<0.001	−0.126	0.071	−0.175	0.012
Uric acid	**−0.217**	0.002	**−0.226**	0.001	−0.193	0.005
Insulin	**−0.398**	<0.001	**−0.318**	<0.001	**−0.253**	<0.001
Fasting glucose	−0.158	0.023	−0.186	0.007	−0.111	0.112
HbA1c	−0.133	0.056	−0.175	0.012	−0.196	0.005
HOMA-R	**−0.393**	<0.001	**−0.321**	<0.001	**−0.245**	<0.001
Hs-CRP	**−0.298**	<0.001	**−0.238**	0.001	−0.168	0.016
Serum Fe	0.068	0.333	0.029	0.680	−0.053	0.449
Ferritin	−0.154	0.028	−0.137	0.051	−0.104	0.139
NO_x_	−0.042	0.550	−0.127	0.068	−0.115	0.101
H_2_O_2_	0.042	0.552	0.061	0.380	0.012	0.869
8-OHdG	0.074	0.293	0.018	0.792	0.041	0.557
8-Isoprostane	0.101	0.149	0.063	0.371	0.172	0.014

Notes: BMI, body mass index; TC, total cholesterol; LDL-c, low-density lipoprotein cholesterol; HDL-c, high-density lipoprotein cholesterol; HbA1c, haemoglobin A1c; HOMA-R, homeostasis model assessment ratio; Hs-CRP, high-sensitivity C-reactive protein; NOx, nitrogen oxide; H_2_O_2_, hydrogen peroxide; 8-OHdG, 8-hydroxy-2’-deoxyguanosine; ^**1**^ Number of cigarette per day; ^**2**^ Drinking unit: 1 unit of alcohol = 9–12g of ethanol; ^**3**^ Serum retinol, α-tocopherol, and γ-tocopherol were adjusted by triglycerides.

**Table 4:** Multiple regression analysis of serum retinol with each parameter.

Explanatory Variable	Serum Retinol ^d^		
	β	*p*	Adjusted R^2^
All subjects **^a^**			0.125
HOMA-R	−0.278	0.001	
Age < 40 **^b^**			0.266
Frequency of alcohol consumption	0.344	0.001	
Insulin	−0.301	0.009	
Age ≥ 40 **^c^**			0.134
HOMA-R	−0.262	0.021	
Ferritin	−0.211	0.032	

Notes: HOMA-R, homeostasis model assessment ratio; All variables are log-transformed; ^**a**^ Adjusted for age, waist circumference, frequency of alcohol consumption, smoking (nonsmoker, smoker), exercise, ferritin, dietary intakes of sodium, niacin, β-carotene, retinol, α-tocopherol, β-tocopherol, γ-tocopherol, δ-tocopherol, vitamin C; ^**b**^ Adjusted for waist circumference, smoking (nonsmoker, smoker), exercise, dietary intakes of sodium, niacin, β-carotene, retinol, α-tocopherol, β-tocopherol, γ-tocopherol, δ-tocopherol, vitamin C; ^**c**^ Adjusted for waist circumference, alcohol consumption, smoking (nonsmoker, smoker), exercise, dietary intakes of β-carotene, retinol, α-tocopherol, β-tocopherol, γ-tocopherol, δ-tocopherol, vitamin C; ^**d**^ Serum retinol was adjusted by triglycerides.

**Table 5:** Multiple regression analysis of serum α-tocopherol with each parameter.

Explanatory Variable	Serum α-Tocopherol ^d^		
	β	*p*	Adjusted R^2^
All subjects **^a^**			0.093
Smoking (nonsmoker, smoker)	−0.160	0.030	
HOMA-R	−0.266	0.001	
Age < 40 **^b^**			0.135
Frequency of alcohol consumption	0.188	0.096	
Age ≥ 40 **^c^**			0.157
Smoking (nonsmoker, smoker)	−0.286	0.008	
HOMA-R	−0.236	0.035	
Ferritin	−0.223	0.022	

Notes: HOMA-R, homeostasis model assessment ratio; All variables are log-transformed; **^a^** Adjusted for age, waist circumference, frequency of alcohol consumption, exercise, ferritin, dietary intakes of sodium, niacin, β-carotene, retinol, α-tocopherol, β-tocopherol, γ-tocopherol, δ-tocopherol, vitamin C; **^b^** Adjusted for waist circumference, smoking (nonsmoker, smoker), exercise, insulin, dietary intakes of sodium, zinc, retinol, α-tocopherol, β-tocopherol, γ-tocopherol, δ-tocopherol, vitamin C; **^c^** Adjusted for waist circumference, alcohol consumption, exercise, insulin, dietary intakes of retinol, α-tocopherol, β-tocopherol, γ-tocopherol, δ-tocopherol, vitamin C; **^d^** Serum α-tocopherol was adjusted by triglycerides.

**Table 6:** Multiple regression analysis of serum γ-tocopherol with each parameter.

Explanatory Variable	Serum γ-Tocopherol ^d^		
	β	*p*	Adjusted R^2^
All subjects **^a^**			0.104
Smoking (nonsmoker, smoker)	−0.161	0.031	
HOMA-R	−0.153	0.065	
8-Isoprostane	0.201	0.006	
NO_x_	−0.151	0.034	
Age < 40 **^b^**			0.121
Frequency of alcohol consumption	0.219	0.041	
HbA1c	−0.274	0.011	
Age ≥ 40 **^c^**			0.070
Smoking (nonsmoker, smoker)	−0.229	0.040	
HOMA-R	−0.224	0.052	

Notes: NO_x_, nitrogen oxide; HbA1c, hemoglobin A1c; HOMA-R, homeostasis model assessment ratio; All variables are log-transformed; ^**a**^ Adjusted for age, waist circumference, frequency of alcohol consumption, exercise, ferritin, dietary intakes of sodium, niacin, β-carotene, retinol, α-tocopherol, β-tocopherol, γ-tocopherol, δ-tocopherol, vitamin C; ^**b**^ Adjusted for waist circumference, smoking (nonsmoker, smoker), exercise, dietary intakes of retinol, α-tocopherol, β-tocopherol, γ-tocopherol, δ-tocopherol, vitamin C; ^**c**^ Adjusted for waist circumference, alcohol consumption, exercise, HOMA-R, dietary intakes of retinol, α-tocopherol, β-tocopherol, γ-tocopherol, δ-tocopherol, vitamin C; ^**d**^ Serum γ-tocopherol was adjusted by triglycerides.

### 3.2. Cigarette Smoking Is Inversely Associated with Circulating Antioxidant Vitamins

We also found that cigarette smoking was inversely associated with the serum α-tocopherol and γ-tocopherol levels (Table 5 and Table 6); these findings are supported by a study by Bruno *et al.* [29], in which a faster disappearance of both plasma α-tocopherol and γ-tocopherol was observed in smokers. However, these reports on the association between smoking and blood tocopherol levels have been contradicted. Some studies [30,31] failed to observe a significant association between smoking and the serum tocopherol levels, whereas Galan *et al.* [32] found that current smokers had significantly lower levels of vitamin E in women, but not in men. It is known that cigarette tar contains high concentrations of ROS [33] and the dose-dependent formation of single-strand breaks in the DNA by extracts of cigarette tar was observed [34]. The present findings suggest that increased oxidative stress in current smokers might slowly drain the antioxidant levels in the circulation.

### 3.3. Alcohol Consumption Positively Associated with Circulating Antioxidant Vitamins

The present findings revealed that the frequency of alcohol consumption was positively associated with the serum retinol, α-tocopherol, and γ-tocopherol levels among the subjects under 40 (Table 4, Table 5 and Table 6); this relationship is consistent with that of previous studies [35,36,37]. However, several studies failed to find any relationship between alcohol consumption and the serum vitamin E levels [32,36]. In chronic alcoholics, reduced levels of plasma retinol and RBP have been observed [38]. In an animal study, an acute dose of ethanol resulted in a marked increase of the serum vitamin A level, possibly due to a decreased uptake by the liver of retinyl esters as part of the lipoproteins [39], whereas in a 4-week human intervention study, the plasma vitamin E and vitamin A levels in the alcohol intake group (0.5 g/kg/day) did not change significantly [40]. The present results describing the association between alcohol consumption and these serum antioxidant vitamins need to be confirmed in future investigations.

### 3.4. Serum Retinol and α-Tocopherol Levels were Inversely Associated with the Serum Ferritin Level

We observed negative relationships between serum ferritin and the retinol and α-tocopherol status in men (Table 3, Table 4 and Table 5). Ferritin in the blood is a marker reflecting the body’s iron stores; it is also regarded as one of the oxidative stress biomarkers because it provides Fe^2+^ for the Fenton reaction [1]. A recent report indicated that men with β-thalassemia had 9.6 times higher levels of serum ferritin, and their serum retinol and α-tocopherol levels were a half and a fourth of the controls, respectively [41]. The previous [1,41] and present findings may suggest that a higher iron status might drain antioxidant vitamins in blood through the production of free radicals.

It has been suggested that the serum α-tocopherol status is inversely related to the risk of prostate cancer, and serum retinol and α-tocopherol levels were found to be lower in patients with cerebellar atrophy [42]. A large population-based study in Sweden [43] revealed a positive association between the total-iron binding capacity and the risk of overall cancer, and increased serum ferritin levels were observed in pre-diabetic subjects [44] and diabetic patients [45]. Taken together, these findings might imply that the consumption of these antioxidant vitamins increases to counteract excessive ROS production in individuals with these diseases.

### 3.5. Study Limitations

The present study had a number of identified limitations: (1) The data were collected only from one prefecture, and the sample size was small; therefore, caution should be taken to avoid generalising the present findings; (2) The lifestyle data, such as the dietary nutrient intake, alcohol consumption, and smoking, relied on self-reporting, which might be subject to recall bias; (3) We employed a FFQ to estimate the nutrient intake. This choice may have weakened the magnitude of the relationship between the dietary nutrients with serum vitamins and clinical biomarkers due to the presence of a fixed list of foods and food groups, variation in interpretation of dietary questions, *etc.* Additionally, the cross-sectional nature of the study indicates that we cannot examine the causality of serum retinol, α-tocopherol and γ-tocopherol with clinical biomarkers among the participants.

## 4. Conclusions

The present study supports an inverse association of serum retinol, α-tocopherol and γ-tocopherol with HOMA-R in healthy men and suggests that the circulatory levels of antioxidant vitamins may modulate the action of insulin. These findings also demonstrate that cigarette smoking was inversely associated with the serum levels of α-tocopherol and γ-tocopherol. A longitudinal design with a large sample size will be necessary in a future study to verify the present results.

## References

[1] Halliwell B., Gutteride J.M.C., Halliwell B., Gutteride J.M.C. (2007). Oxidative Stress. Free Radicals in Biology and Medicine.

[2] Acheson R.M., Williams D.R. (1983). Does consumption of fruit and vegetables protect against stroke?. Lancet.

[3] Gey K.F., Moser U.K., Jordan P., Stähelin H.B., Eichholzer M., Lüdin E. (1993). Increased risk of cardiovascular disease at suboptimal plasma concentrations of essential antioxidants: An epidemiological update with special attention to carotene and vitamin C. Amer. J. Clin. Nutr..

[4] (1997). Food, Nutrition and the Prevention of Cancer: A Global Perspective.

[5] Diplock A.T., Machlin L.J., Packer L., Proyer W.A. (1989). Vitamin E: Biochemistry and health implications. Ann. N. Y. Acad. Sci..

[6] Palan P.R., Mikhail M.S., Goldberg G.L., Basu J., Runowicz C.D., Romney S.L. (1996). Plasma levels of beta-carotene, lycopene, canthaxanthin, retinol, and alpha- and tau-tocopherol in cervical intraepithelial neoplasia and cancer. Clin. Cancer Res..

[7] Mizuno Y., Furusho T., Yoshida A., Nakamura H., Matsuura T., Eto Y. (2006). Serum vitamin A concentrations in asthmatic children in Japan. Pediatr. Int..

[8] Persson C., Sasazuki S., Inoue M., Kurahashi N., Iwasaki M., Miura T., Ye W., Tsugane S., JPHC Study Group (2008). Plasma levels of carotenoids, retinol and tocopherol and the risk of gastric cancer in Japan: A nested case-control study. Carcinogenesis.

[9] Whitney E.N., Rolfes S.R., Whitney E.N., Rolfes S.R. (2002). The Fat-soluble Vitamins: A, D, E, and K. Understanding Nutrition.

[10] Rózanowska M., Cantrell A., Edge R., Land EJ., Sarna T., Truscott T.G. (2005). Pulse radiolysis study of the interaction of retinoids with peroxyl radicals. Free Radic. Biol. Med..

[11] Behrens W.A, Madère R. (1986). Alpha- and gamma tocopherol concentrations in human serum. J. Amer. Coll. Nutr..

[12] Kamal-Eldin A., Appelqvist L.A. (1996). The chemistry and antioxidant properties of tocopherols and tocotrienols. Lipids.

[13] Jiang Q., Christen S., Shigenaga M.K., Ames B.N. (2001). Gamma-tocopherol, the major form of vitamin E in the USA diet, deserves more attention. Amer. J. Clin. Nutr..

[14] Sato Y., Ogino K., Sakano N., Wang D.H., Yoshida J., Akazawa Y., Kanbara S., Inoue K., Kubo M., Takahashi H. (2013). Evaluation of urinary hydrogen peroxide as an oxidative stress biomarker in a healthy Japanese population. Free Radic. Res..

[15] Iwanaga S., Sakano N., Taketa K., Takahashi N., Wang D.H., Takahashi H., Kubo M., Miyatake N., Ogino K. (2013). Comparison of serum ferritin and oxidative stress biomarkers between Japanese workers with and without metabolic syndrome. Obes. Res. Clin. Pract..

[16] Driskell W.J., Lackey A.D., Hewett J.S., Bashor M.M. (1985). Stability of vitamin A in frozen sera. Clin. Chem..

[17] Gunter E.W., Driskell W.J., Yeager P.R. (1988). Stability of vitamin E in long-term stored serum. Clin. Chim. Acta.

[18] Takahashi K., Yoshimura Y., Kaimoto T., Kunii D., Komatsu T., Yamamoto S. (2001). Validation of a food frequency questionnaire based on food groups for estimating individual nutrient intake. Jpn. J. Nutr..

[19] Willett W.C., Howe G.R., Kushi L.H. (1997). Adjustment for total energy intake in epidemiologic studies. Amer. J. Clin. Nutr..

[20] Hussain M.M. (2000). A proposed model for the assembly of chylomicrons. Atherosclerosis.

[21] Salonen J.T., Nyyssönen K., Tuomainen T.P., Mäenpää P.H., Korpela H., Kaplan G.A., Lynch J., Helmrich S.P., Salonen R. (1995). Increased risk of non-insulin dependent diabetes mellitus at low plasma vitamin E concentrations: A four year follow up study in men. BMJ.

[22] Kamimura W., Doi W., Takemoto K., Ishihara K., Wang D.H., Sugiyama H., Oda S.I., Masuoka N. (2013). Effect of vitamin E on alloxan-induced mouse diabetes. Clin. Biochem..

[23] Houstis N., Rosen E.D., Lander E.S. (2006). Reactive oxygen species have a causal role in multiple forms of insulin resistance. Nature.

[24] Blondin S.A., Yeung E.H., Mumford S.L., Zhang C., Browne R.W., Wactawski-Wende J., Schisterman E.F. (2013). Serum retinol and carotenoids in association with biomarkers of insulin resistance among premenopausal women. ISRN Nutr..

[25] Kawaguchi R., Yu J., Honda J., Hu J., Whitelegge J., Ping P., Wiita P., Bok D., Sun H. (2007). A membrane receptor for retinol binding protein mediates cellular uptake of vitamin A. Science.

[26] Craft N.E. (2001). Innovative approaches to vitamin A assessment. J. Nutr..

[27] Quadro L., Blaner W.S., Salchow D.J., Vogel S., Piantedosi R., Gouras P., Freeman S., Cosma M.P., Colantuoni V., Gottesman M.E. (1999). Impaired retinal function and vitamin A availability in mice lacking retinol-binding protein. EMBO J..

[28] Yang Q., Graham T.E., Mody N., Preitner F., Peroni O.D., Zabolotny J.M., Kotani K., Quadro L., Kahn B.B. (2005). Serum retinol binding protein 4 contributes to insulin resistance in obesity and type 2 diabetes. Nature.

[29] Bruno R.S., Ramakrishnan R., Montine T.J., Bray T.M., Traber M.G. (2005). α-Tocopherol disappearance is faster in cigarette smokers and is inversely related to their ascorbic acid status. Amer. J. Clin. Nutr..

[30] Comstock G.W., Menkes M.S., Schober S.E., Vuilleumier J.P., Helsing K.J. (1988). Serum levels of retinol, beta-carotene, and alpha-tocopherol in older adults. Amer. J. Epidemiol..

[31] Bolton-Smintch C., Casey C.E., Gey K.F., Bolton-Smith C., Smith W.C., Tunstall-Pedoe H. (1991). Antioxidant vitamin intakes assessed using a food-frequency questionnaire: Correlation with biochemical status in smokers and non-smokers. Br. J. Nutr..

[32] Galan P., Viteri F.E., Bertrais S., Czernichow S., Faure H., Arnaud J., Ruffieux D., Chenal S., Arnault N., Favier A., Roussel A.M., Hercberg S. (2005). Serum concentrations of beta-carotene, vitamins C and E, zinc and selenium are influenced by sex, age, diet, smoking status, alcohol consumption and corpulence in a general French adult population. Eur. J. Clin. Nutr..

[33] Pryor W.A., Stone K. (1993). Oxidants in cigarette smoke. Radicals, hydrogen peroxide, peroxynitrate, and peroxynitrite. Ann. N. Y. Acad. Sci..

[34] Borish E.T., Cosgrove J.P., Church D.F., Deutsch W.A., Pryor W.A. (1985). Cigarette tar causes single-strand breaks in DNA. Biochem. Biophys. Res. Commun..

[35] Herbeth B., Chavance M., Musse N., Mejean L., Vernhes G. (1989). Dietary intake and other determinants of blood vitamins in an elderly population. Eur. J. Clin. Nutr..

[36] Talwar D., McConnachie A., Welsh P., Upton M., O’Reilly D., Smith G.D., Watt G., Sattar N. (2010). Which circulating antioxidant vitamins are confounded by socioeconomic deprivation? *The MIDSPAN family study*. PLoS One.

[37] Woodside J.V., Young I.S., Gilchrist S.E., Vioque J., Chakravarthy U., de Jong P.T., Rahu M., Seland J., Soubrane G., Tomazzoli L., Topouzis F., Vingerling J.R., Fletcher A.E. (2013). Factors associated with serum/plasma concentrations of vitamins A, C, E and carotenoids in older people throughout Europe: The EUREYE study. Eur. J. Nutr..

[38] McClain C.J., van Thiel D.H., Parker S., Badzin L.K., Gilbert H. (1979). Alterations in zinc, vitamin A, and retinol-binding protein in chronic alcoholics: A possible mechanism for night blindness and hypogonadism. Alcohol. Clin. Exp. Res..

[39] Sato M., Lieber C.S. (1982). Changes in vitamin A status after acute ethanol administration in the rat. J. Nutr..

[40] Suzukawa M., Ishikawa T., Yoshida H., Hosoai K., Nishio E., Yamashita T., Nakamura H., Hashizume N., Suzuki K. (1994). Effects of alcohol consumption on antioxidant content and susceptibility of low-density lipoprotein to oxidative modification. J. Amer. Coll. Nutr..

[41] Behera S., Dixit S., Bulliyya G., Kar S.K. (2013). Fat-soluble antioxidant vitamins, iron overload and chronic malnutrition in β-thalassemia major. Indian J. Pediatr..

[42] Weinstein S.J., Wright M.E., Lawson K.A., Snyder K., Männistö S., Taylor P.R., Virtamo J., Albanes D. (2007). Serum and dietary vitamin E in relation to prostate cancer risk. Cancer Epidemiol. Biomark. Prev..

[43] Gaur A., Collins H., Wulaningsih W., Holmberg L., Garmo H., Hammar N., Walldius G., Jungner I., van Hemelrijck M. (2013). Iron metabolism and risk of cancer in the Swedish AMORIS study. Cancer Cause. Control.

[44] Sharifi F., Nasab N.M., Zadeh H.J. (2008). Elevated serum ferritin concentrations in prediabetic subjects. Diab. Vasc. Dis. Res..

[45] Kim C.H., Kim H.K., Bae S.J., Park J.Y., Lee K.U. (2011). Association of elevated serum ferritin concentration with insulin resistance and impaired glucose metabolism in Korean men and women. Metabolism.

